# Removal of Toxin (Tetrodotoxin) from Puffer Ovary by Traditional Fermentation

**DOI:** 10.3390/toxins5010193

**Published:** 2013-01-18

**Authors:** Kensaku Anraku, Kiku Nonaka, Toshitaka Yamaga, Takatoshi Yamamoto, Min-Chul Shin, Masahito Wakita, Ayaka Hamamoto, Norio Akaike

**Affiliations:** 1 Research Division for Life Science, Kumamoto Health Science University, 325 Izumi-machi, Kitaku, Kumamoto 861-5598, Japan; E-Mails: anraku@kumamoto-hsu.ac.jp (K.A.); nonaka@kumamoto-hsu.ac.jp (K.N.); tyamaga@kumamoto-hsu.ac.jp (T.Y.); yamataka@kumamoto-hsu.ac.jp (T.Y.); karosu94@hanmail.net (M.-C.S); wakitamasahito@gmail.com (M.W.); hamamoto@kumamoto-hsu.ac.jp (A.H.); 2 Research Division for Clinical Pharmacology, Medical Corporation, JuryoGroup, Kumamoto Kinoh Hospital, 6-8-1 Yamamuro, Kitaku, Kumamoto 860-8518, Japan

**Keywords:** puffer fish, *Takifugu Stictonotus*, fermented puffer’s ovary, tetrodotoxin, rat hippocampal neuron, sodium current

## Abstract

The amounts of puffer toxin (tetrodotoxin, TTX) extracted from the fresh and the traditional Japanese salted and fermented “*Nukazuke*” and “*Kasuzuke*” ovaries of *Takifugu stictonotus* (*T. stictonotus*) were quantitatively analyzed in the voltage-dependent sodium current (*I*_Na_) recorded from mechanically dissociated single rat hippocampal CA1 neurons. The amount of TTX contained in “*Nukazuke*” and “*Kasuzuke*” ovaries decreased to 1/50–1/90 times of that of fresh ovary during a salted and successive fermented period over a few years. The final toxin concentration after fermentation was almost close to the TTX level extracted from *T. Rubripes”* fresh muscle that is normally eaten. It was concluded that the fermented “*Nukazuke*” and “*Kasuzuke*” ovaries of puffer fish *T. Stictonotus* are safe and harmless as food.

## 1. Introduction

Esculent puffer fish’s fresh muscle and skin contains less toxin (TTX) compared to the liver and ovary. Thus, they are usually eaten as Japanese traditional puffer fish dishes such as “*Sashimi*”, “*Tessa*”, “*Tecchiri*” and are also deep fried. However, in the Hokuriku district (Shirayama city, Ishikawa Prefecture) in Japan, there are traditional salted and fermented food named “*Nukazuke*” or “*Kasuzuke*” which use virulent ovaries: *i.e.*, ovaries plus rice bran or Sake lees for a year and half to two years after salting then down for six months to a year, that is for two years to three years in total. By this treatment, the toxicity of ovary decreased remarkably so that it could be served as food [1,2].

**Figure 1:**
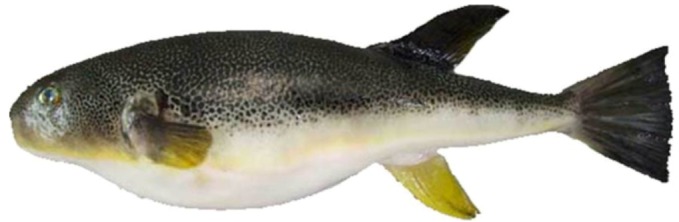
Puffer fish *Takifugu stictonotus (T. stictonotus)*. The muscle and ovary extracts were prepared for electrophysiological bioassay of tetrodotoxin (TTX).

Until now, we have reported on the toxin content of not only cultured puffer fish *Takifugu rubripes* (*T. rubripes*) but also other four wild puffer fishes that live in the sea, near the coast of Japan [3,4]. Since the sodium channel is selectively blocked by TTX [5,6], the TTX amounts of muscle, liver, ovary, kidney, intestine and eyes were also examined quantitatively in voltage-dependent sodium currents (*I*_Na_) by using mechanically isolated rat hippocampal CA1 pyramidal neurons [3]. Thus, the aim of this study is to survey the TTX amount contained in two kinds of fermented ovary food sold in markets as “*Nukazuke*” and “*Kasuzuke*” on *I*_Na_ by using whole-cell patch recording configuration. The TTX amounts in fresh and fermented *T. Stictonotus*’ (Figure 1) ovaries were compared in order to consider the safety of the fermented puffer fish’s ovary as food.

## 2. Results

### 2.1. Effects of Toxin Extracts from the Muscle and Ovary of Puffers on I_Na_

**Figure 2:**
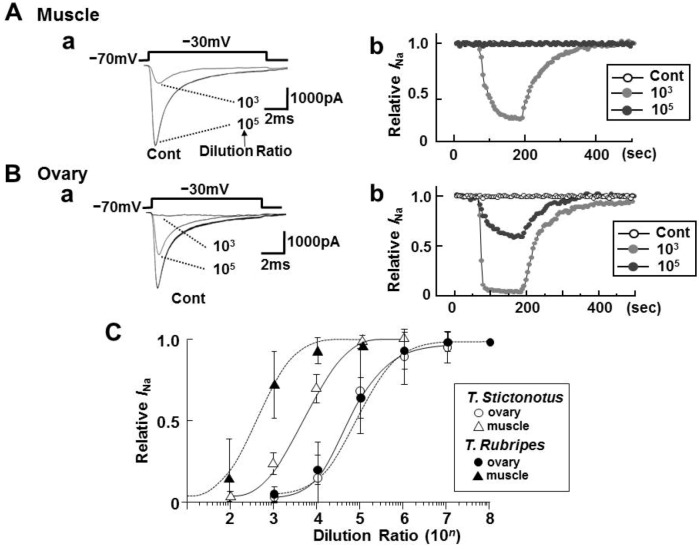
(**A**, **B**) Effects of toxin extracts from fresh muscle and ovary T. Stictonotus on voltage-dependent sodium current (*I*_Na_) of isolated rat hippocampal CA1 neurons. *I*_Na_ was evoked by depolarizing step pulse from a holding potential (*V*_H_) of −70 mV to −30 mV every 5 s. The toxin extracts were diluted to 10^2^–10^6^ times for muscle and 10^3^–10^8^ times for ovary. Aa and Ba are representable current traces with and without toxin extracts from muscle and ovary diluted 10^3^ and 10^5^ times, respectively. Ab and Bb are time courses of *I*_Na_ inhibition and washout effect with and without the toxin extracts from muscle and ovary of *T. stictonotus*, respectively; (**C**) Dilution-inhibition curves for toxin extracts from fresh muscles and ovaries from *T. stictonotus* and *T. rubripes*. Each point is the mean ± SD of 6–9 neurons.

*I*_Na_ was evoked by a depolarized step pulse with 10 ms duration in dissociated CA1 pyramidal neurons of rats from a holding potential (*V*_H_) of −70 mV to −30 mV. Figure 2Aa,Ba show representative *I*_Na_ traces with and without toxin extracts from ovary and muscle of wild *T. Stictonotus*, respectively. In the presence of these toxin extract, *I*_Na_ was inhibited in a time- and concentration-dependent fashion. The rapid inhibition and slow washout effect as seen in Figure 2Ab,Bb were also observed with tetrodotoxin alone (TTX, Sigma) as reported in our previous studies [3,4,6].

Figure 2C indicates the dilution-inhibition curves for the toxin extracts from fresh muscles and ovaries of the two kinds of puffer fishes (*T. stictonotus* and *T. rubripes*). The strength of the toxin estimated from IC_50_ values of the dilution-inhibition curves was in the order of *T. stictonotus* 3.8 × 10^3^ Dil > *T. Rubripes* 1.7 × 10^2^ Dil for muscles, and *T. stictonotus* 4.4 × 10^4^ Dil ≈ *T. rubripes* 5.7 × 10^4^ Dil for ovaries.

### 2.2. Effects of Toxin Extracts from *“Nukazuke”* and *“Kasuzuke”* Ovaries of *T. Stictonotus*

**Figure 3:**
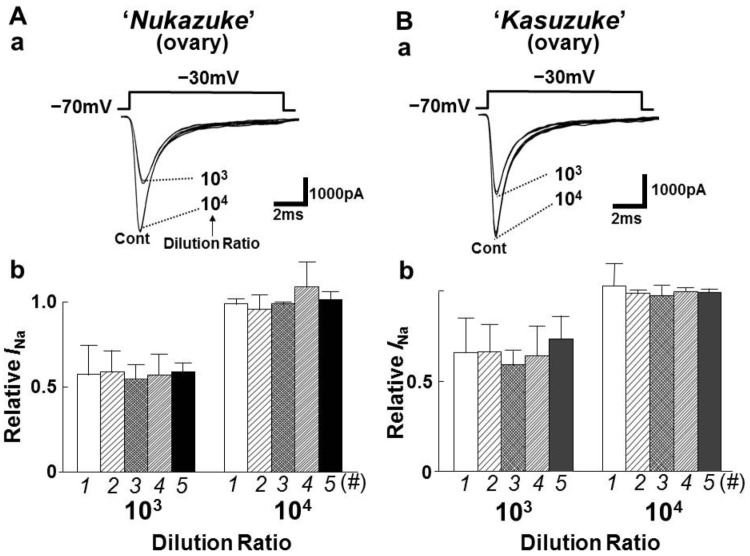
Effects of toxin extracts from the salted and fermented “*Nukazuke*” (**A**) and “*Kasuzuke*” (**B**) ovaries of *T. Stictonotus* on *I*_Na_. Aa and Ba are typical currents traces of *I*_Na_ with and without 10^3^−10^4^ times diluted “*Nukazuke*” and “*Kasuzuke*” extracts, respectively. Ab and Bb present the inhibition of *I*_Na_ by toxin extracts (10^3^ or 10^4^ Dil) obtain from four different “*Nukazuke*” and “Kasuzuke” ovaries (#1–5), respectively. X axis shows the data from five different ovaries of “*Nukazuke*” or “*Kasuzuke*”. Y axis is relative *I*_Na_. Each column is mean ± SD of 6–8 neurons.

**Figure 4:**
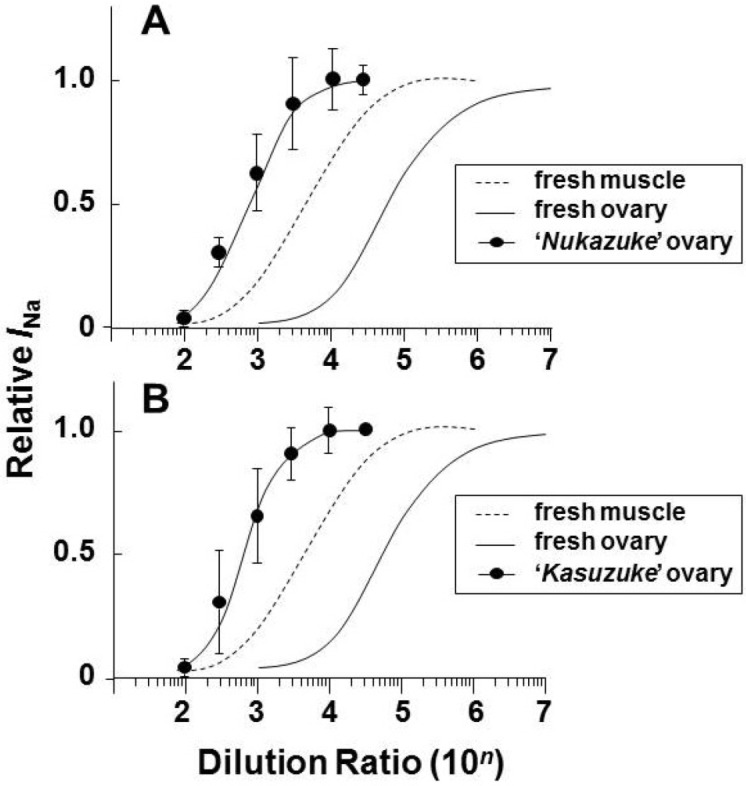
Dilution-inhibition curves for toxin extracts from “*Nukazuke*” (**A**) and “*Kasuzuke*” (**B**) ovaries. Data of fresh muscle and ovary of *T. stictonotus* (dotted and straight line curves, respectively) were quoted from Figure 2. Each point is the mean ± SD of 6–9 neurons.

The toxin extracts of the salted and fermented “*Nukazuke*” and “*Kasuzuke*” ovaries were diluted at six different concentrations between 10^2^ and 3 × 10^4^ times and they were examined on *I*_Na_. Figure 3Aa and Ba show the representative current traces in the presence of 10^3^ or 10^4^ diluted “*Nukazuke*” and “*Kasuzuke*” ovary extracts on *I*_Na_. Figure 3Ab,Bb represent the inhibitory variation of *I*_Na_ with and without 10^3^ and 10^4^ diluted toxin extracts from each five “*Nukazuke*” and “*Kasuzuke*” ovaries. It is evident in the figure that the five ovaries of both “*Nukazuke*” and “*Kasuzuke*” contained almost similar amounts of toxin, suggesting that there are little variation in the TTX amounts among individuals of “*Nukazuke*” or “*Kasuzuke*” ovaries. Figure 4A,B summarize the dilution-inhibition curves induced by toxin extracts prepared from “*Nukazuke*” ovaries (A) and “*Kasuzuke*” ovaries (B) of *T. stictonotus,* where both dotted and solid line curves were quoted from the dilution-inhibition curves of fresh muscle and ovary of *T. stictonotus* (Figure 2C).

The inhibitory values calculated from IC_50_ values of the respective dilution-inhibition curves (Figure 4) of “*Nukazuke*” and “*Kasuzuke*” ovaries and fresh ovary were 9.4 × 10^2^ Dil for “*Nukazuke*” ovary, and 5.0 × 10^2^ Dil for “*Kasuzuke*” ovary. The obtained diluted values are quite small as compared with 4.4 × 10^4^ Dil for fresh ovary, suggesting that the toxin amount of *T. stictonotus* ovaries decreased markedly during a long salted and fermentation period.

### 3.3. Estimated Toxin Amount in the Fresh and Fermented Ovaries of *T. stictonotus*

**Figure 5:**
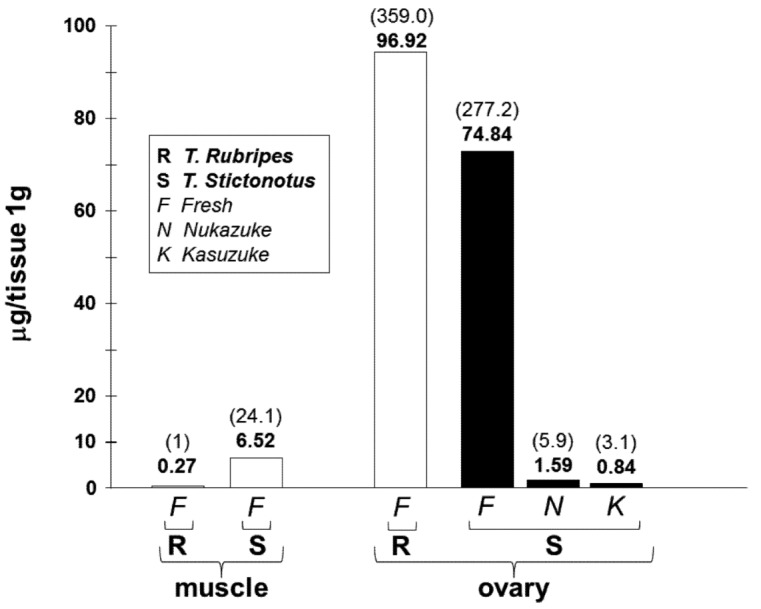
The amount of toxin extracts expressed as μg TTX/g tissue in fresh muscle, fresh ovaries, salted and fermented “*Nukazuke*” and “*Kasuzuke*” ovaries. Data were obtained from *T. stictonotus* and *T. rubripes*. The number in brackets is the ratio of toxin amount in muscles and ovaries to fresh muscle of *T. rubripes*.

The toxin extracts of puffers selectively suppressed *I*_Na_ of rat hippocampal CA1 neurons as reported in present and previous reports [3,4]. Thus it is possible to estimate the toxin amount from toxin extracts with and without the salted and fermented treatments of *T. stictonotus*’ ovaries as an amount of equipotent TTX by comparing IC_50_ values of the toxin extracts. Figure 5 summarizes the mean value of toxin content expressed as the amount of microgram TTX/1g tissue in the “*Nukazuke*” and “*Kasuzuke*” ovaries of *T. stictonotus* and the fresh muscles and ovaries of both *T. rubripes* and *T. stictonotus*. The toxin amounts of two kinds of puffer fresh muscles are also shown in Figure 5 (left panel). The amount of TTX in muscles was 24 times higher in *T. stictonotus* than in *T. rubripes* while that in ovaries was only 1.3 times higher in *T. stictonotus* than in *T. Rubripes*. Surprisingly, the toxin level (74.8 μg/g) of fresh ovary of *T. slictonotus* decreased markedly to about 1/50–1/90 times in “*Nukazuke*” (1.59 μg/g) and “*Kasuzuke*” (0.84 μg/g) ovaries, respectively. In addition, the toxin amount of salted and fermented ovaries of *T. stictontous* became less than that (6.52 μg/g) of the fresh muscles. The toxin level of these fermented ovaries was almost close to *T. rubripes*’ fresh muscle (0.27 μg/g).

## 3. Discussions

The reduction of the amount of puffer toxin (TTX) in salted and fermented *T. Stictonotus*’ “*Nukazuke*” or “*Kasuzuke*” ovaries has been determined by mice IC_50_ test. According to Ozawa’s whole animal (mouse) studies [1,2], TTX amount of fresh ovaries reduced from 450 MU/g to 100 MU/g during half to one year salting down, continue reducing to 10 MU/g through two years of “*Nukazuke*” or “*Kasuzuke*” fermentation process and eventually less than 10 MU, at which MU value these fermented ovaries are edible, as permitted by the Japanese government. In the present electrophysiological measurements, the amount of TTX decreased from 74.84 μg/g for fresh ovary of *T. stictonotus* to 1.59 μg/g for “*Nukazuke*” ovary or 0.84 μg/g for “*Kasuzuke*” ovary. Although the reduced TTX amount of these salted and fermented ovaries is about 3–6 times higher than esculent *T. Rubripes*’ muscle (0.27 μg/g), it is still a harmless amount to eat. Therefore, present electrophysiological findings support the consequence determined from the MU measurement by Ozawa [2].

The reduction of TTX caused by bacteria in the medium soaked puffer fish “*Nukazuke*” and “*Kasuzuke*” ovaries was reported by Kobayashi *et al.* [7,8]. According to these reports, although several strains were isolated from the “*Nukazuke*” and “*Kasuzuke*” which showed TTX reduction in the medium, the reduction was attributed to the alkalization of the incubation medium coupling with the amplification of bacteria. TTX is dissolved in weak acid solution but not dissolved in neutral solution. Therefore, it is considered that TTX is concentrated in liver and ovary containing a high ratio of lipid. At this time, it has been concluded that the mechanism of TTX reduction in “*Nukazuke*” and “*Kasuzuke*” ovaries is caused by the salting-out lipid group containing TTX in saturated NaCl condition followed by preserving in the rice bran and sake lees, but not by breakdown of TTX by bacteria [1,8].

In our previous study, the TTX amount of *T. rubripes*’ ovary and liver contained about 350 times greater than that of muscle [3]. In generally, *T. rubripes*’ muscle contained the lowest TTX amount (0.25–0.27 μg/1 g tissue), which is about 1/400 of ovary and liver. However, *T. stictonotus*’ muscle contained relatively high TTX amount (6.52 μg/g) as comparing those of other puffer fish muscles (*T. ruburipes*, 0.25 μg/g, *L. wheeleri*, 0.35 μg/g, *C. rivulata*, 0.25 μg/g *A. reticularis*, 0.19 μg/g except *T. vermicularis*, 8.43 μg/g) [4]. Thus, TTX amount (6.52 μg/g) of *T. Stictonotus*’ muscle is close to *T. vermicularis*’ one (8.43 μg/g). Including these studies, The organs from both *T. ruperipes* and *T. Stictonotus* are generally less toxic than organs from other species like *T. poecilonotus*, *T. snyderi*, *T. flavidus* [9]. The latter species were inedible unlike as a fresh muscle of *T. ruperipes*.

In the report by Ikeda *et al.* [10], the enormous seasonal changes in toxicity of puffer fish organs including ovary, liver and skin were observed. The liver toxicity was high during the ordinary period, and that of ovary was high during the maturation period. Skin toxicity in general was maintained throughout the year, but fell significantly during the maturation period, though the fluctuation range was much smaller than that of liver toxicity. In all three tissues, it should be noted that toxicity declined markedly just after spawning. The ovary for the use of “*Nukazuke*” and “*Kasuzuke*” were generally obtained from Japan Sea Coast from May to June, and toxicity in the ovary was highest during this season. Nevertheless, the final toxin concentration after fermentation was almost close to the TTX level extracted from *T. rubripes*’ fresh muscle that is normally eaten. Thus, the traditional fermentation method is an effective way to reduce the TTX amount in ovaries. According to the inspection of Ishikawa prefecture, there is no puffer fish poisoning incidents of “*Nukazuke*” and “*Kasuzuke*”.

## 4. Experimental Section

### 4.1. Puffers

In the present experiments, we used two different puffers, namely *T. Rubripes* (2.0–2.5 kg) and *T. Stictonotus* (0.6–1.0 kg) (Figure 1). Female puffers of *T. Rubripes* were captured in the Sea of Genkai, Fukuoka Prefecture, Kyushu and *T. Stictonotus* in the Sea of Japan, Ishikawa Prefecture, Honshu, Japan. “*Nukazuke*” and “*Kasuzuke*” were purchased from Bokuchin Co., Ltd. (Ishikawa, Japan).

### 4.2. Toxin Extract from the Fresh Muscle, and the Fresh, *“Nukazuke”* and *“Kasuzuke”* Ovaries of Puffers

Toxin extracts were prepared according to the method described elsewhere [3,4,11]. The reagents for extraction, methanol, acetic acid, diethyl ether, and citric acid were purchased from Wako Pure Chemical Industries, Ltd. (Osaka, Japan). Briefly, tissues including muscle and ovary were excised from each puffer, transferred in an ice-cold box, transported to the laboratory and kept frozen at −20 °C. The toxin extracts were made from fresh muscle or ovary pieces (20 g each) prepared from six *T. Rubripes* and twenty *T. Stictonotus*. In the case of “*Nukazuke*” or “*Kasuzuke*”, the toxin extracts were made from ovary pieces (20 g each) prepared from four *T. Stictonotus,* which have already had extraneous matter removed by hands wearing a latex glove. These frozen samples (20 g each) were homogenized with a small amount of 2% acetic acid-methanol (*v*/*v*). The derived suspension was extracted with 50 mL of 2% acetic acid-methanol (*v*/*v*) under refluxing conditions for 10 min, and centrifuged at 2500 rpm for 15 min at room temperature (21–25 °C). The supernatant was filtered through a Kiriyama funnel. The filtrate was evaporated under reduced pressure, and the sediments were combined for a second cycle of extraction. Each extraction was carried out three times. The residue was diluted with purified water (15 mL), and the solution was extracted with diethyl ether (15 mL) three times to remove the hydrophobic component. The water phase was evaporated under reduced pressure and the residue, diluted with 7.5 mL citrate buffer (pH 4.80), was kept frozen below −20 °C.

In each electrophysiological experiment, the citrated stock solution of toxin extract was diluted by external solution which contained (in mM) NaCl 60, choline-Cl 100, CsCl 5, CaCl_2_ 2.5, LaCl_3_ 0.01, TEA-Cl 5, glucose 10, HEPES 10 and pH was adjusted to 7.40 with Tris-base at room temperature. All test solutions containing drugs were applied by “Y-tube system” for rapid solution exchange within 20 ms [4].

### 4.3. Cell Preparation

All experiments were performed in accordance with the Guiding Principles for Care and Use of Laboratory Animals in The Japanese Pharmacological Society and approved by the Local Animal Experiment Committee in Kumamoto Health Science University.

CA1 pyramidal neurons were isolated from the dorsal site of the rat hippocampus. Wistar rats (12–18 days postnatal; KYUDO, Kumamoto, Japan) were anesthetized with pentobarbital sodium (50 mg/kg i.p.) and then decapitated. Hippocampi were removed and cut into fine transverse slices (400 μm) with a vibrating slice cutter (VT1200S; Leica, Nussloch, Germany) in a ice-cold incubation solution (in mM) NaCl 124, KCl 5, KH_2_PO_4_ 1.2, MgSO_4_ 1.3, CaCl_2_ 2.4, NaHCO_3_ 24 and glucose 10, bubbled with 95% O_2_ and 5% CO_2_. Cutting slices containing the CA1 region were recovered in incubation solution saturated well with 95% O_2_ and 5% CO_2_ at room temperature (21–25 °C) for at least 1 h before mechanical dissociation. Mechanically dissociated CA1 neurons were prepared as reported previously [12]. In brief, a slice was transferred into a 15 mm culture dish (Primaria 3801; Becton Dickinson, Rutherford, NJ, USA) containing standard solution (in mM) NaCl 150, KCl 5, MgCl_2_ 1, CaCl_2_ 2, glucose 10 and HEPES 10, pH adjusted to 7.40 with Tris-base at room temperature and the CA1 region was identified under a binocular microscope (SMZ645; Nikon, Tokyo, Japan). Mechanical dissociation was accomplished with a fire-polished glass pipette coupled to a vibration device (SI-10 Cell Isolator; K.T. Labs, Tokyo, Japan). The tip of the glass pipette was lightly placed on the surface of the CA1 region and vibrated horizontally (0.3–0.5 mm displacement) at about 50–60 Hz. These dissociated neurons maintained their original morphological features with proximal dendrites of 50–100 μm. Within 30 min, the neurons attached to the bottom of the Petri dish and were used for electrophysiological recordings.

### 4.4. Sodium Current Recording

The *I*_Na_ was recorded in the whole-cell configuration by the use of a conventional patch-clamp technique [3]. Neurons were viewed under phase contrast using an inverted microscope (DMIRB; Leica, Nussloch, Germany). Macroscopic *I*_Na_ was measured using a patch-clamp amplifier (Multiclamp 700B; Molecular Devices, Sunnyvale, CA, USA). Patch pipettes were made from borosilicate capillary glass (1.5 mm outer diameter, 0.9 mm inner diameter, G-1.5; Narishige, Tokyo, Japan) in two stages on a vertical pipette puller (PP-830; Narishige, Tokyo, Japan). The pipette solution (in mM) was CsF 105, NaF 30, CsCl 5, TEA-Cl 5, EGTA 2 and HEPES 10, pH adjusted to 7.2 with Tris-base. The electrodes filled with the solution had a tip resistance of 3–4 МΩ. All experiments were performed at room temperature (21–25 °C). All membrane currents were filtered at 2 kHz with a low-pass filter (E-3201A Decade Filter; NF Electronic Instruments, Tokyo, Japan), and stored on a computer using pCLAMP 10.2 (Axon Instruments, Foster City, CA, USA). 10 mV hyperpolarizing step pulses (30 ms duration) were used to monitor the access resistance. If the access resistance of neurons changed by more than 20%, the recording was rejected. Neurons were voltage-clamped at a holding potential (*V*_H_) of −70 mV throughout the experiments. All experiments were carried out at room temperature to compare with our previous report [13].

### 4.5. Statistical Analysis

The relative *I*_Na_ was calculated by the following equation:

Relative *I*_Na_ = *I*/*I*_0_ (1)

where *I* is the peak *I*_Na_ in standard solution containing TTX (Sigma-Aldrich, Tokyo, Japan) or extracted toxin, and *I*_0_ is the peak *I*_Na_ in standard solution without TTX. Dose-inhibition curves were fitted with Origin Software 7.5 (OriginLab, Northampton, MA, USA) to a sigmoidal dose response equation for IC_50_ value determination. The potencies of the toxin extract to inhibit *I*_Na_ were expressed as an amount of equipotent TTX, comparing the IC_50_ obtained from dilution-inhibition curve and dose-inhibition curve for TTX. In our previous study [3], IC_50_ was calculated for the relative *I*_Na_ at concentrations between 0.1 and 100 nM standard curve for TTX (Sigma-Aldrich, Tokyo, Japan). IC_50_ value on the peak *I*_Na_ was 14.1 nM. Thus, the amount of toxin (TTX/1.0 g of each tissue) was estimated by comparing IC_50_ of the toxin extracts to that of TTX (Sigma-Aldrich, Tokyo, Japan). Data were presented as mean ± standard deviation (SD). 

## 5. Conclusions

The present results indicated that the amount of TTX contained in “*Nukazuke*” (1.59 μg/g) and “*Kasuzuke*” (0.84 μg/g) ovaries decreased to 1/50–1/90 times of that of fresh ovary (74.84 μg/g) during a salted and successive fermented period of a few years. The final toxin concentration after fermentation was almost close to the TTX level extracted from *T. Rubripes*’ fresh muscle that is normally eaten. It was concluded that the fermented “*Nukazuke*” and “*Kasuzuke*” ovaries of puffer fish *T. Stictonotus* are safe and harmless as food.
